# A Primary Urothelial Carcinoma Presenting as a Mid-Bulbar Urethral Stricture in a 30-Year-Old Male

**DOI:** 10.7759/cureus.16774

**Published:** 2021-07-31

**Authors:** Mohammad A Alghafees, Raouf M Seyam, Waleed M Altaweel, Omar S Alghamdi, Turki Al-Hussain, Tariq Alotaibi, Abdullah Alturki, Laila Alessa

**Affiliations:** 1 Medicine, King Saud Bin Abdulaziz University for Health Sciences, Riyadh, SAU; 2 Urology, King Faisal Specialist Hospital and Research Centre, Riyadh, SAU; 3 Medicine, King Saud University, Riyadh, SAU; 4 Pathology, King Faisal Specialist Hospital and Research Centre, Riyadh, SAU; 5 Urology, King Saud Medical City, Riyadh, SAU

**Keywords:** urethral stricture, high grade urothelial carcinoma, uro oncology, rare cancers, surgical oncology

## Abstract

The bulbar urethra is the most common site of anterior urethral strictures. In this case report, we present a 30-year-old male who was referred to us as a case of mid-bulbar urethral stricture. Urethroplasty was booked and a papillary lesion was found on routine diagnostic cystoscopy. An open biopsy was taken which showed invasive high-grade papillary urothelial carcinoma with squamous differentiation. This case is rare in terms of a young age of incidence, a lack of risk factors, an absence of suspicious symptoms, and a short duration of signs and symptoms. Urologists should consider workup for malignancy even in young patients who present with an idiopathic urethral stricture and a short duration of symptoms.

## Introduction

Transitional cells line the urinary system, which runs from the renal pelvis via the ureters, bladder, and urethra, as well as the prostatic ducts in males. Although it is most commonly seen in the bladder, urothelial carcinoma (UC), also known as transitional cell carcinoma (TCC), is high-grade cancer that can develop in any region of the urinary system with a high proclivity for spreading [1,2]. It is a phenotypically diverse tumor with a broad range of morphological characteristics as well as a plethora of molecular changes and subtypes.

Of the urethral bulb strictures, around 40%, in the industrialized world, are idiopathic with some reports suspecting an underlying congenital etiology [3]. Reports in the literature also established instrumentation, particularly in hypospadias surgery, trauma, and a history of sexually transmitted infections as prominent risk factors of bulbar urethral strictures [3-5]. Bulbomembranous urethral tumors usually have a late detection. However, when treated at early stages, their prognosis is usually favorable [6].

In this case, we report a 30-year-old man who was referred as a case of urethral stricture and was eventually diagnosed as a case of UC.

## Case presentation

A medically free 30-year-old Saudi male was referred to our setup as a case of urethral stricture. Six months ago, he had gastric sleeve surgery. Since then, he started to have recurrent attacks of urinary retention, a weak urinary stream, and difficulty in urinating. He was not suffering from any suspicious symptoms, such as hematuria and recurrent urinary tract infections. On history acquisition, the patient was not a smoker, had no significant history of trauma, exposure to radiation and chemicals, sexually transmitted diseases, or tumors. He also had no family history of cancers.

**Figure 1:**
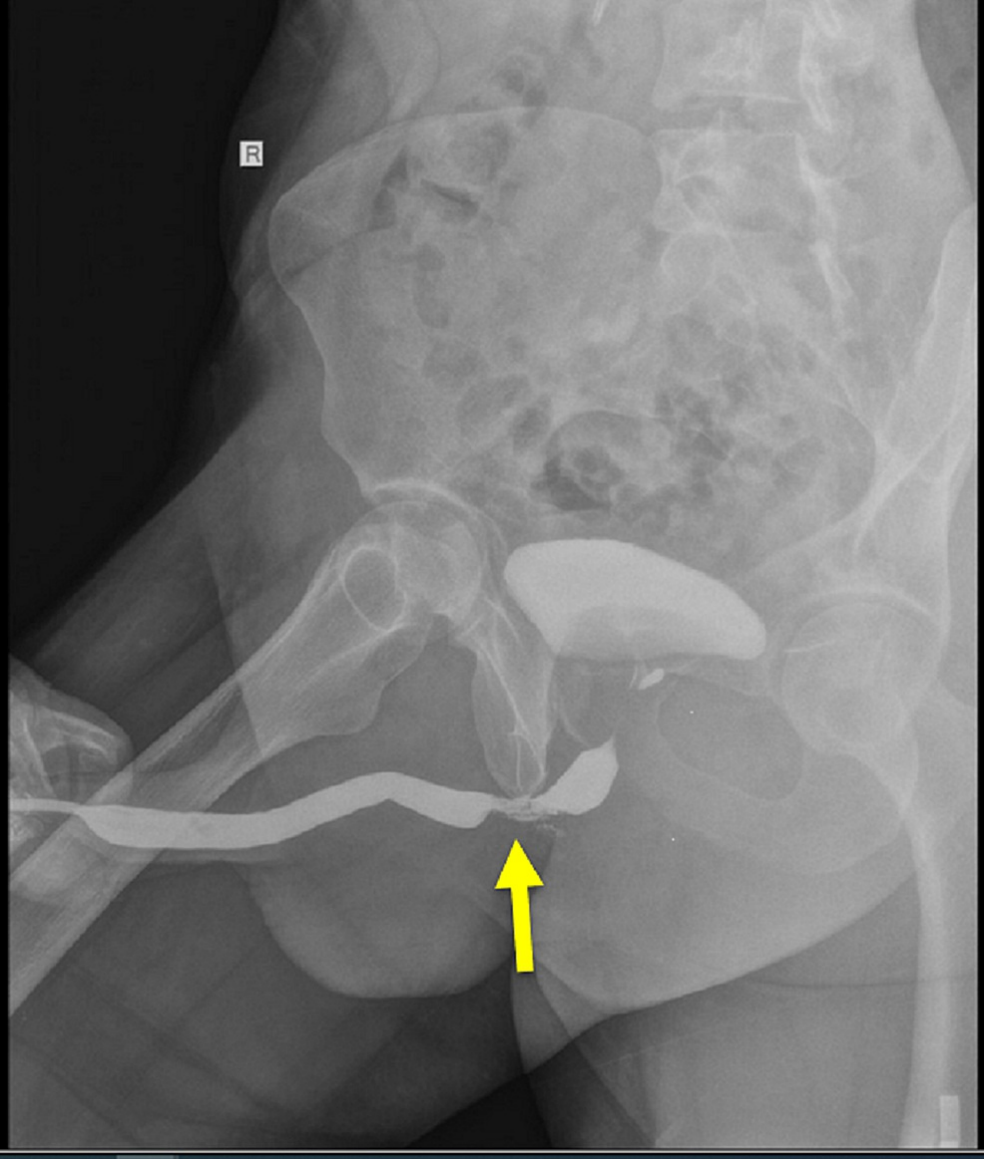
An ascending urethrogram showing a 2-cm short annular stricture segment (yellow arrow) at the bulbourethra with no filling defect.

He had undergone multiple cystoscopies and urethral dilation procedures with the last one being an optical urethrotomy four months ago, but he kept re-stricturing and his symptoms persisted. His latest ascending urethrogram showed a short annular stricture segment measuring 2 cm at the bulbourethra with no filling defect (Figure 1).

**Figure 2:**
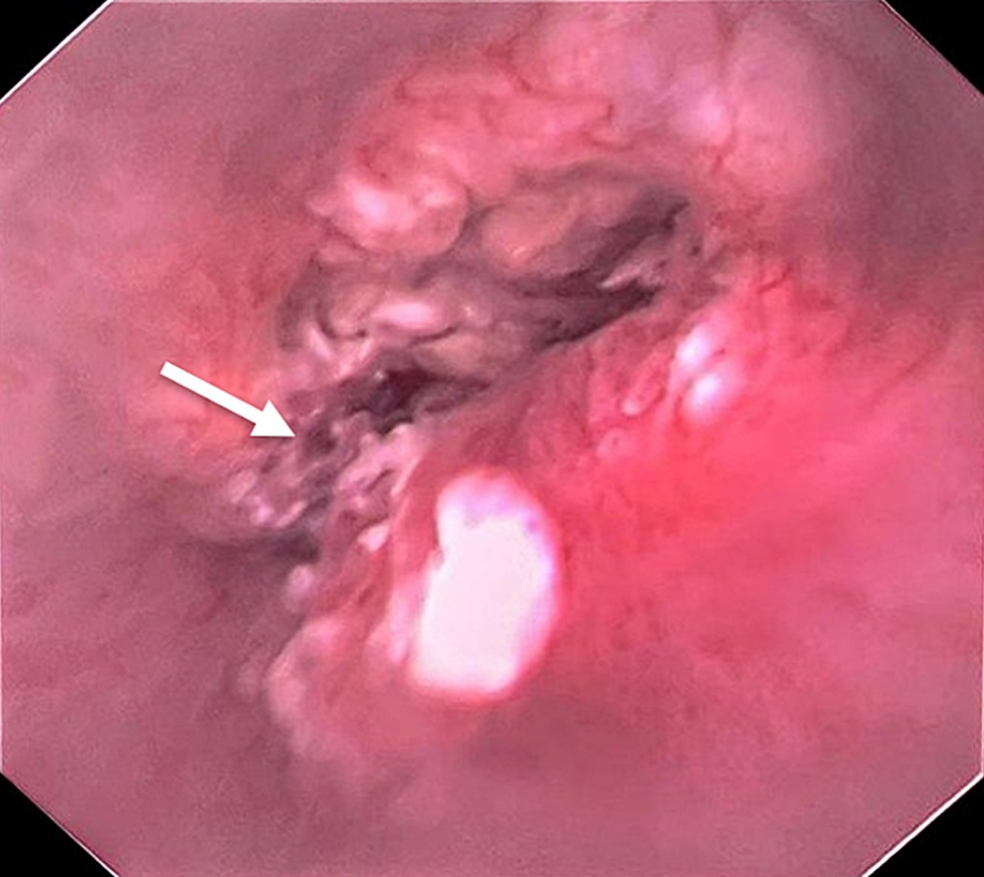
An intraoperative urethroscopy image displaying the papillary lesion (white arrow) found during the attempted urethroplasty.

On examination, he was active and alert with no distress. He had a body mass index (BMI) of 23.7 kg/m^2^. Both his cardiovascular and respiratory examinations were normal. The abdomen was soft, non-tender, and non-distended. His baseline examinations and laboratory tests were all unremarkable. The patient was booked for a urethroplasty. During the routine diagnostic cystoscopy, a papillary lesion in the stricture area was detected (Figure 2).

**Figure 3:**
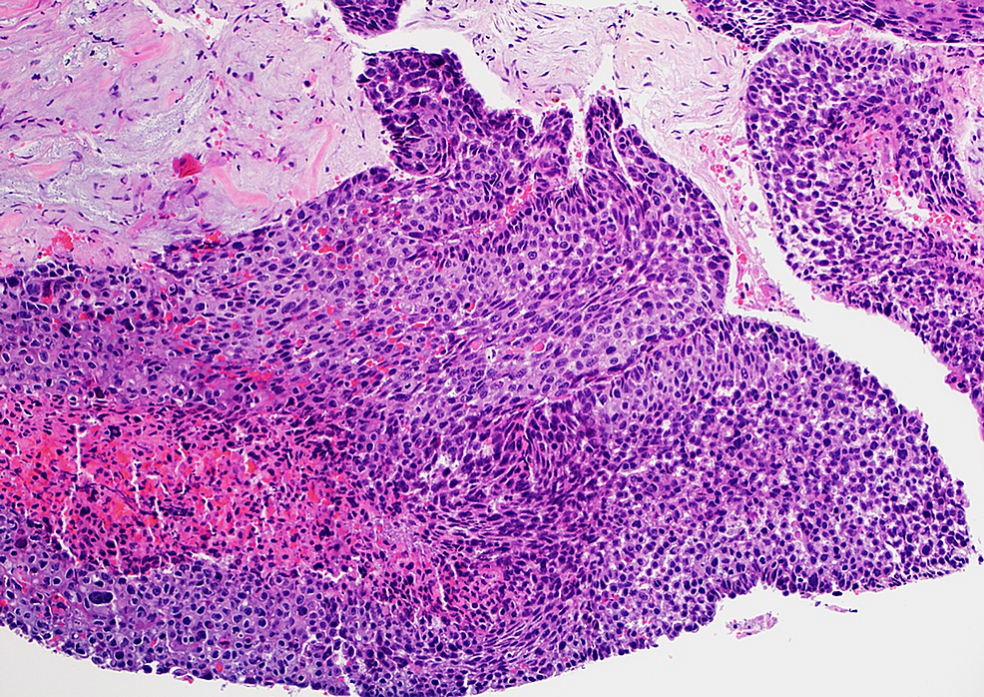
Postoperative pathological examination showing an invasive high-grade papillary urothelial carcinoma (hematoxylin and eosin staining).

**Figure 4:**
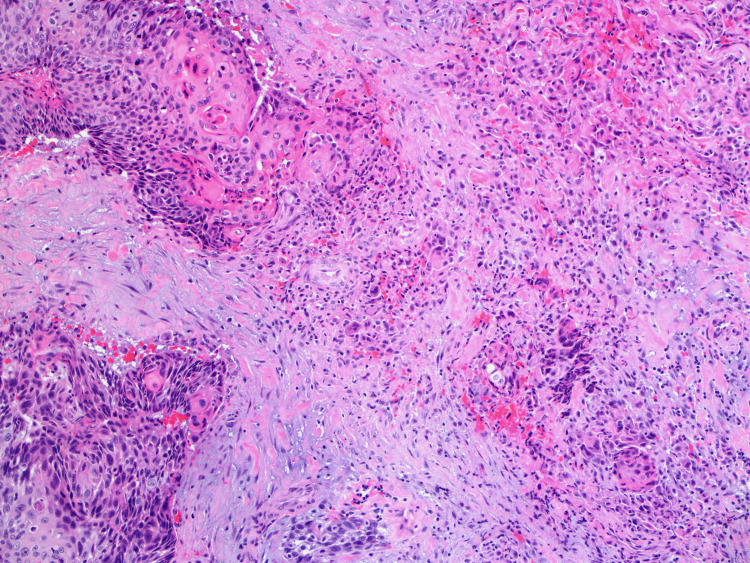
Postoperative pathological examination showing the squamous differentiation and lamina propria invasion of the tumor (hematoxylin and eosin staining).

An open biopsy from both the spongiosa and the urethral mucosa was obtained by preforming an incision in the urethra and performing dissection. The procedure was aborted, and a cystodilation was done instead. The biopsy report showed invasive high-grade papillary urothelial carcinoma with squamous differentiation and lamina propria invasion (Figures 3, 4).

A contrast-enhanced CT scan of the chest, abdomen, and pelvis was done and showed no local invasion, lymph node involvement, or distant metastasis. Only benign sclerotic rims in the right iliac bone were found. An in situ hybridization (ISH) was done and showed no evidence for human papillomavirus (HPV) infection. The treatment plan was concluded to be radical cystoprostatectomy along with urethrectomy as it is currently the curative gold standard.

## Discussion

This is a rare occurrence of UC of the urethra presenting as a case of bulbourethral stricture in a young patient. Typically, UC of the urethra occurs predominantly during the sixth decade of life, and about 55% to 65% occur in the bulbar urethra. The mean age of diagnosis is 69 years among men and 71 years among women [7]. UCs are uncommon in young adults with fewer than 1% of those tumors presenting during the first four decades of life [8]. Its incidence rates are highest in males, elderly patients, and African Americans [9]. The most common subtype is the urothelial [9].

The presentation of urethral cancer in our patient is unique. The patient was young, had no risk factors for urothelial cancer, and had a short history of the presentation related to recent surgery and catheterization. Many factors could have predisposed this patient to primary UC. These include genetic predisposition and hormone receptor abnormalities. Studies show that upregulation of the p53 gene is frequent in young individuals with urothelial cancer [10]. However, the clinical behavior, recurrence of diseases, and advancement in young patients with a diagnosis of urothelial cancer are still a place of debate. A substantial quantity of clinical evidence suggests that a major element of urothelial carcinogenesis and tumor development are the steroid hormone receptor-mediated signals. These receptors include androgen receptors, estrogen receptors, progesterone receptors, as well as glucocorticoid receptors. Investigations have shown that increased or decreased receptors expression or changes in their pathways are linked to patient outcomes. Steroid hormone receptors and associated signals can therefore be used as biomarkers of UCs and can feasibly predict tumor recurrence or progression [11].

The early presentation of primary UC is commonly a lower urinary obstructive symptom that mimics a stricture of the urethra [9]. The presence of risk factors or symptoms suggesting cancer like hematuria, or a rapid course of symptoms may suggest a more sinister pathology. The delayed presentation may include physical signs such as a urethral mass or evidence of metastasis [12]. The clinical picture of our patient did not raise the suspicion of the correct diagnosis because of the absence of suggestive symptoms or signs, the young age, and the multiple urethral stricture treatments he received elsewhere in a short period.

In the literature, there have been a few similar cases to ours. For instance, Raphael et al. reported a case of anterior urethral carcinoma which presented as a urethral stricture. The patient’s initial symptoms were similar to the presented case, i.e., urinary retention [13]. Similarly, Gupta et al. reported a 33-year-old male with primary adenocarcinoma of the bulbomembranous urethra, which had spread to the prostatic urethra and left inguinal lymph nodes [14]. Moreover, Shah et al. described a squamous cell carcinoma in 78 years old in the anterior urethra. Similarly, the patient presented with a slow and interrupted urinary stream. However, it was accompanied by hematuria, urge incontinence, nocturia, and a urinary infection persisting for over a year before presentation. It is noteworthy to mention that the tumor was markedly immunoreactive for p16, a surrogate marker of HPV, which is an established risk factor of squamous cell carcinoma [15]. Furthermore, in an American study involving 130 primary urethral tumors, of which 106 were carcinomas, a synchronous HPV infection was documented 31.65% of the cases. Akin to our presented case, most of the tumors had squamous and urothelial features. However, the patient in our presented case did not have any evidence of sexually transmitted diseases including HPV [16].

Males with UC should be evaluated by a detailed history, examination under anesthesia, and radiological imaging of the pelvis, abdomen, and chest [17]. However, no definite guidelines have been made for the treatment of the UC of the urethra due to its rarity. Treatment is individualized and depends on the location and staging of cancer [18]. Extensive radical surgical removal of the tumor is currently regarded as the mainstay of management. However, small tumors of the urethra have been proven to be able to be treated with transurethral resection and penile preserving surgery [19-21]. A level one evidence could not be established to support the efficacy of chemotherapy and radiotherapy in the management of UC of the urethra due to the rarity of such conditions.

## Conclusions

This is a unique case of a young male with UC presenting as a case of mid-bulbar urethral stricture with a rare age of incidence, a lack of risk factors, the absence of suspicious symptoms, and short signs and symptoms duration. Urologists should keep in mind the possibility of a more sinister etiology behind idiopathic strictures of the urethra presenting at a young age with a short duration of symptoms and no history of trauma or infections as it could be the initial presentation of a carcinoma. The management of primary urethral cancer in a young patient is a challenge. Treatment should be individualized and depends on the location, staging of cancer, and patient preference. Further reporting of such cases could be a gateway for more discussion about the clinical behavior, recurrence, and tumor advancement in young patients with a diagnosis of primary UC.
